# The CaSm (LSm1) oncogene promotes transformation, chemoresistance and metastasis of pancreatic cancer cells

**DOI:** 10.1038/oncsis.2015.45

**Published:** 2016-01-11

**Authors:** E C Little, E R Camp, C Wang, P M Watson, D K Watson, D J Cole

**Affiliations:** 1 Department of Microbiology and Immunology, Medical University of South Carolina, Charleston, SC, USA; 2 Hollings Cancer Center, Medical University of South Carolina, Charleston, SC, USA; 3 Department of Surgery, Medical University of South Carolina, Charleston, SC, USA; 4 Ralph H Johnson VA Medical Center, Charleston, SC, USA; 5 Department of Pathology and Laboratory Medicine, Medical University of South Carolina, Charleston, SC, USA; 6 Department of Biochemistry and Molecular Biology, Medical University of South Carolina, Charleston, SC, USA

## Abstract

The cancer-associated Sm-like (CaSm) oncogene is overexpressed in 87% of human pancreatic tumor samples and CaSm knockdown has demonstrated therapeutic efficacy in murine models of pancreatic cancer. Evidence indicates that CaSm modulates messenger RNA degradation; however, its target genes and the mechanisms by which CaSm promotes pancreatic cancer remain largely unknown. Here, we demonstrate that the CaSm overexpression alters several hallmarks of cancer—including transformation, proliferation, chemoresistance and metastasis. Doxycycline-induced CaSm expression enhanced proliferation and both anchorage-dependent and -independent growth of the human Panc-1 cells *in vitro*. CaSm induction decreased gemcitabine-induced cytotoxicity and altered the expression of apoptotic regulation genes, including Bad, E2F1 and Bcl-X_L_. CaSm-overexpressing Panc-1 cells were twofold more migratory and fourfold more invasive than the driver controls and demonstrated characteristics of epithelial-to-mesenchymal transition such as morphological changes and decreased E-cadherin expression. CaSm induction resulted in changes in RNA expression of metastasis-associated genes such as MMP1, SerpinB5, uPAR and Slug. Using a murine model of metastatic pancreatic cancer, injection of CaSm-induced Panc-1 cells resulted in a higher abundance of hepatic metastatic lesions. Overall, CaSm overexpression contributed to a more aggressive cancer phenotype in Panc-1 cells, further supporting the use of CaSm as a therapeutic target against pancreatic cancer.

## Introduction

Pancreatic adenocarcinoma (PC) is the fourth leading cause of cancer-related deaths in the nation. Owing to its aggressive nature and difficult diagnosis, the majority of patients present with metastatic disease and generally demonstrate poor response to conventional chemotherapy and radiation.^1^ For 15 years, gemcitabine has been considered the best standard-of-care chemotherapeutic agent in the management of PC, prolonging survival about 6 months.^2^ New combinatorial therapeutics, most notably FOLFIRNOX, can substantially improve survival rates, but severe toxicity often limits their clinical use.^3^ Although gemcitabine is generally well tolerated, an analysis of 21 phase III clinical trials, involving over 3000 patients from 1997 to 2011, documented that the overall objective response rate to gemcitabine therapy was a disappointing 8.3%.^4^ Incremental improvement in these response rates with gemcitabine-based combinatorial chemotherapeutic approaches has been encouraging;^5, 6^ however, the lack of recent breakthroughs in treatment highlights the need for new therapeutic strategies. Given that most patients present with more advanced disease, and in the face of recent evidence suggesting that dissemination occurs even before primary pancreas tumor formation,^7^ further research is necessary to investigate what drives metastasis in this disease.

The cancer-associated Sm-like oncogene (CaSm) has been shown to be overexpressed in nearly 90% of human pancreatic tumor samples as well as several other cancer histopathologies.^8^ Also identified as human LSm1, CaSm is a key member in the LSm1–7 complex where it forms a heteromeric ring with six other members of the LSm family. Given its location in cytoplasmic foci along with decapping enzymes Dcp1/2 and exonuclease Xrn1, this LSm complex is hypothesized to facilitate 5′–3′ degradation of cytoplasmic messenger RNA (mRNA).^9, 10, 11, 12, 13^ Consistent with this hypothesis, previous studies have shown that increased CaSm levels results in multiple genetic alterations—including apoptotic genes, several signaling pathways and cell cycle mediators.^14, 15^

Our laboratory has investigated the potential use of CaSm as a therapeutic target and shown that CaSm expression is necessary for cancer formation in mice.^16^ Furthermore, decreasing CaSm expression in a murine PC model resulted in decreased *in vivo* tumor formation and increased survival,^16, 17^ whereas combinatorial antisense CaSm and gemcitabine therapies additively enhanced survival in a subcutaneous PC mouse model.^18^ In short, CaSm is a promising target for a difficult cancer that has had few new therapies developed in the past decade.

Beyond the LSm1 correlates, relatively little remains known about the role for CaSm in the process of neoplastic transformation or how its downregulation in the setting of established neoplasia leads to decreased tumor growth. To explore this further, we developed models that would allow the induction of CaSm overexpression in a PC cell line intrinsically expressing relatively low CaSm levels. Here, we demonstrate that induced CaSm expression results in increased growth, decreased chemotherapeutic sensitivity and enhanced migration/invasion in PC cells. CaSm upregulation alters the gene expression of critical mediators of apoptosis, metastasis and epithelial-to-mesenchymal transition (EMT), which complements the proposed function for CaSm in mRNA regulation and provides a putative mechanism for CaSm-mediated neoplastic progression.

## Results

### Induction of CaSm levels in human Panc-1 cells

**Figure 1:**
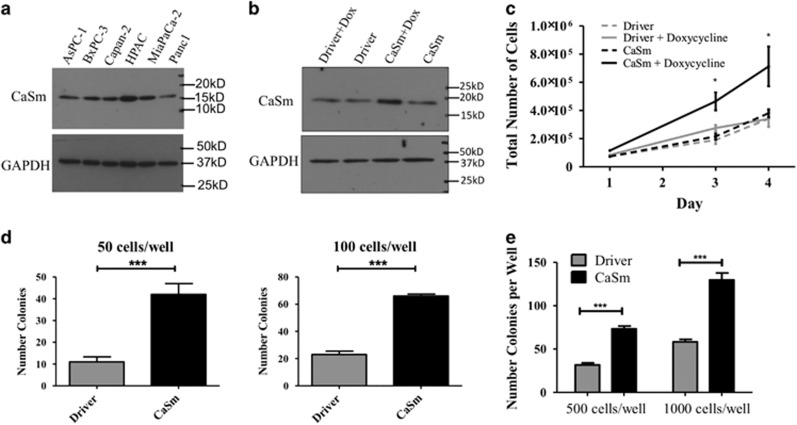
CaSm induction enhances the proliferation and transformation of Panc-1 cells. (**a**) CaSm expression was analyzed in human PC cell lines using western blot analysis with GAPDH as a control. (**b**) Tet-on driver and tet-on CaSm Panc-1 cells were grown in the absence or presence of 1 μg/ml doxycycline for 24 h before assessed for CaSm expression by western blot analysis with GAPDH as a control. (**c**) In all, 100 000 tet-on driver and tet-on CaSm Panc-1 cells were plated in triplicate in six-well plates on day 0. Cell proliferation was quantified with Trypan blue cell counts on days 1, 3 and 4. Depicted is a representative of three independent experiments with mean cell count and s.e.m. shown. (**d**) Tet-on driver and tet-on CaSm Panc-1 cells were plated at 50 cells per well or 100 cells per well in triplicate in six-well plates with media containing 1 μg/ml doxycycline. After 2 weeks, cells were stained with 0.01% crystal violet and colonies containing at least 50 cells quantified. The mean quantification and s.e.m. are depicted. (**e**) Tet-on driver and tet-on CaSm cells were plated at 500 or 1000 cells per well in 0.4% agar in six-well plates. In all, 0.4% agar containing growth media and 1 μg/ml doxycycline was added once a week. After 5 weeks, cells were stained with 0.1% crystal violet in 10% ethanol and quantified by counting the number of resulting colonies at least 0.1 mm in diameter. Depicted is a representative of three independent experiments with mean cell count and s.e.m. shown. *P<0.05, ***P<0.001.

CaSm protein expression was evaluated via western blot analysis in a panel of human PC cell lines to identify a cell line with lower levels of basal expression. CaSm expression was variable among the cell lines, with Panc-1 cells demonstrating the lowest endogenous levels (Figure 1a). Owing to their low endogenous CaSm expression, Panc-1 cells were selected for gain-of-function studies to better identify the molecular consequences of CaSm upregulation. Panc-1 cells were stably transfected with the Clontech RetroX system to create a doxycycline-induced CaSm pool (tet-on CaSm) with tet-on driver Panc-1 cells as a control (Figure 1b). CaSm expression was induced in a doxycycline dose-dependent manner (Supplementary Figure 1a) with greatest induction achieved with 1 μg/ml doxycycline, which was used in all subsequent experiments. Induced CaSm expression reached maximum levels 8–12 h after addition of 1 μg/ml doxycycline (Supplementary Figure 1b).

### Induced CaSm overexpression promotes growth and transformation in Panc-1 cells

Previous studies demonstrated that adenoviral-delivered antisense CaSm RNA reduced Panc-1 cell growth by 44%.^18^ Reciprocally, induced CaSm expression significantly enhanced cellular growth twofold compared with driver and non-induced controls (Figure 1c). Low-density clonogenic assays identified that CaSm-overexpressing cells formed colonies more readily compared with driver controls. At 100 cells per well, induced CaSm expression resulted in a nearly threefold increase in colony count (66±2.6 colonies in the tet-on CaSm Panc-1 compared with 23±4.4 colonies in the driver controls; Figure 1d). These differences were even more pronounced at lower plating densities of 50 cells per well, where CaSm-induced cells produced four times the number of colonies (42±8.7 compared with 11±4; Figure 1d), indicating that CaSm produced a transformed cellular phenotype with less dependence on cellular density and cytokine interactions. Furthermore, induced CaSm upregulation enhanced the Panc-1 colony formation in soft agar. After 5 weeks of incubation in 0.4% soft agar, the tet-on CaSm Panc-1 cells established twice the number of colonies than the driver control cells at both 500 and 1000 cells per well (Figure 1e), providing further evidence that CaSm has a significant role in transformation and cancer development.

### CaSm induction results in chemoresistance to gemcitabine *in vitro*

**Figure 2:**
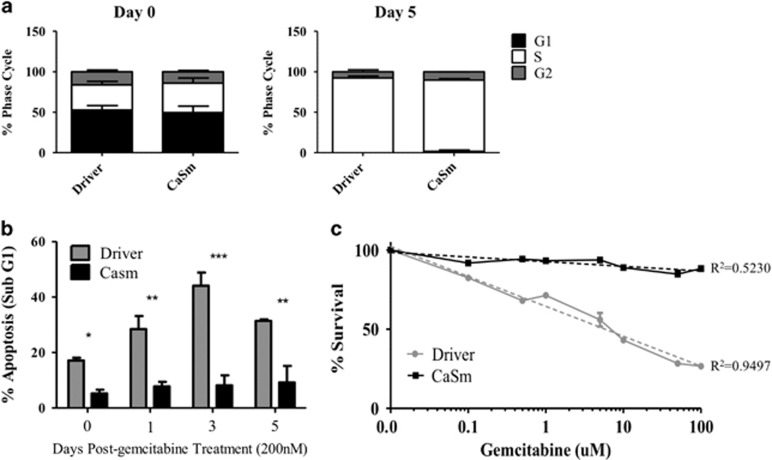
CaSm overexpression protects Panc-1 cells from gemcitabine-induced cytotoxicity. (**a**, **b**) Tet-on driver and tet-on CaSm Panc-1 cells were grown in 1 μg/ml doxycycline before being treated with 200 nM gemcitabine on day 0. Cells were trypsinized and fixed in ice-cold ethanol on days 0, 1, 3 and 5, stained with 0.1 μg/μl PI and analyzed for cell cycle content by flow cytometry analysis. (**a**) Cell cycle distribution on days 0 and 5, (**b**) depicts quantification of mean sub-G1 content on individual days along with s.e.m., (**c**) Tet-on driver or CaSm Panc-1 cells were seeded in each well of a 96-well plate (5000 cells per well) overnight. Cells were then treated with 0–100 μM gemcitabine with replicates of at least six wells per condition. Ninety-six hours after treatment, cells were fixed with 5% trichloroacetic acid, stained with 0.4% SRB, and dissolved in 10 mM Tris base before absorbance was read at 560 nm. Depicted is a representative of three independent experiments, along with nonlinear regression (y=linear, x=logarithmic) and respective *R*^2^ values. *P<0.05, **P<0.005, ***P<0.001.

We previously established that decreasing CaSm levels using antisense RNA additively enhances the antitumor effects of gemcitabine therapy both *in vitro* and *in vivo*.^18^ Therefore, we hypothesized that CaSm overexpression would reduce gemcitabine sensitivity. To evaluate this, tet-on driver and CaSm Panc-1 cells were treated with 200 nM gemcitabine and analyzed by flow cytometry cell cycle analysis on days 0, 1, 3 and 5. By day 5, both cell lines were observed to have a nearly complete S phase arrest (Figure 2a). However, the sub-G1 population, which was used to distinguish apoptotic cells as a result of DNA fragmentation, identified a significantly higher percentage of apoptosis in the driver control cell line at all time points compared with the tet-on CaSm cells (Figure 2b). Seventy-two hours after gemcitabine treatment, the percentage of apoptotic cells in the driver control cells reached a peak of 44% apoptosis compared with 8.19% in the tet-on CaSm cells, suggesting that CaSm overexpression protected these PC cells from gemcitabine-induced apoptosis. Sulforhodamine B (SRB) assay, which measures drug-induced cytotoxicity through colorimetric analysis of cellular density,^19^ was further used to determine the response to increasing amounts of gemcitabine. Although driver cells responded to increased gemcitabine with decreased cell viability, the CaSm-overexpressing cells demonstrated significant gemcitabine resistance at all drug concentrations up to 100 μM. Comparing absorbance to their respective untreated controls, the tet-on driver Panc-1 cells demonstrated 26.5% survival after treatment with 100 μM gemcitabine although the tet-on CaSm cells maintained 88.3% viability at this concentration (Figure 2c). Using nonlinear regression, the IC_50_ of the driver cell line was achieved at 5.81 μM gemcitabine, whereas the CaSm-induced cells did not reach 50% lethality with doses up to 100 μM (Figure 2c).

### CaSm overexpression alters apoptotic gene expression

**Figure 3:**
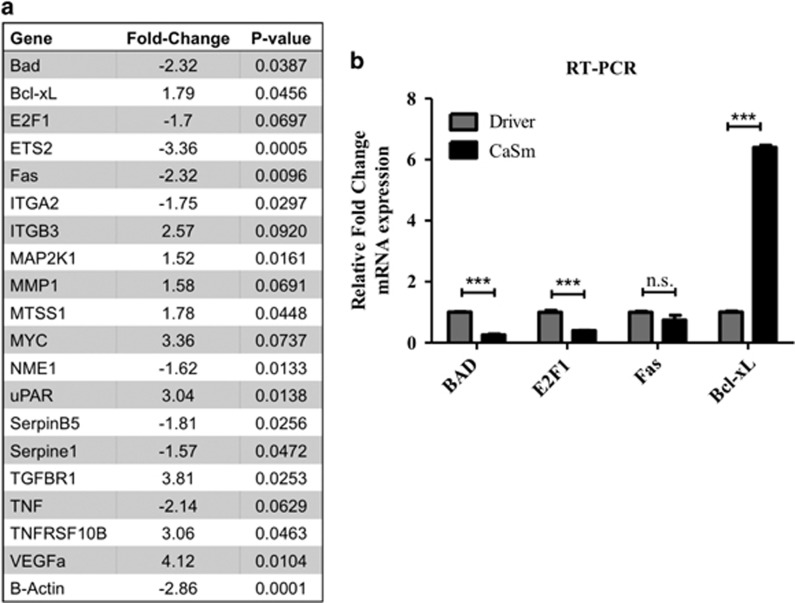
CaSm induction alters apoptotic gene expression. (**a**) Tet-on driver and tet-on CaSm Panc-1 cells were maintained in 1 μg/ml doxycycline for 4 weeks before RNA expression was analyzed by the SABioscience Cancer PathwayFinder PCR-based array. Genes with a 1.5-fold or greater alteration comparing means of two experimental arrays with two driver+dox controls are depicted. (**b**) Real-time PCR was used to validate the microarray with GAPDH used as a control. ***P<0.001.

Given its known role in mRNA stability, we next investigated whether CaSm alters message overexpression in the Panc-1 cells. PCR-based microarray analysis identified several messages with at least a 1.5-fold change in gene expression following CaSm induction compared with driver controls (Figure 3a). Several apoptotic gene messages previously associated with drug resistance demonstrated altered expression—notably, Bcl-X_L_, Bad and E2F1. CaSm overexpression resulted in a 1.79-fold increase in Bcl-X_L_ RNA and a 2.32- and 1.7-fold decrease in Bad and E2F1, respectively, as compared with tet-on driver controls (Figure 3a); these alterations were confirmed by real-time reverse transcriptase–PCR (Figure 3b). Similar changes in gene expression were observed when comparing tet-on Casm Panc-1 cells with both driver and non-induced controls (Supplementary Figure 3). Microarray analysis also identified a 2.32-fold decrease in Fas expression following CaSm induction, which was not validated upon subsequent real-time PCR validation. Changes in expression of these apoptotic genes were consistent with our observations showing an increased resistance to apoptosis induction and provide a possible explanation to gemcitabine chemoresistance.

### CaSm induction enhanced cellular migration and invasion and metastatic gene expression

**Figure 4:**
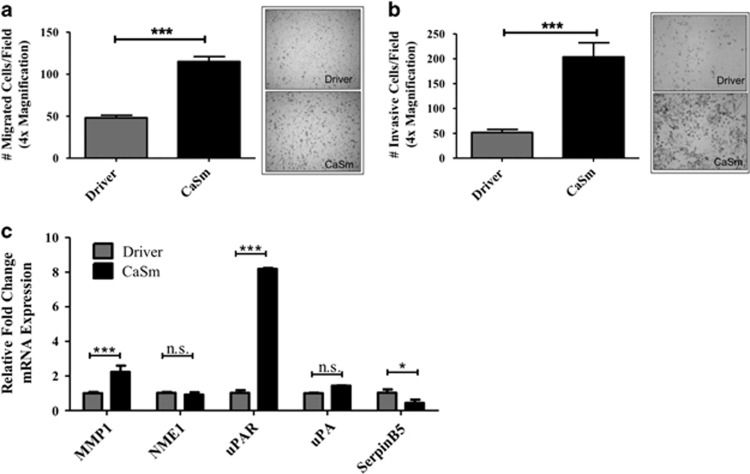
CaSm induction promotes migration and invasion and alters metastatic gene expression. Tet-on driver and tet-on CaSm Panc-1 cells, previously grown in 1 μg/ml doxycycline for 4–8 weeks, were plated in serum-free DMEM onto the top chamber of fibronectin-coated transwell membranes (**a**) or rehydrated Matrigel transwell chambers (**b**). DMEM supplemented with 10% FBS was used as the chemoattractant in the bottom wells. The chambers were incubated at 37 °C for 4 h (**a**) and 48 h (**b**) before transwell cells were stained and quantified by counting random fields at × 40 magnification. Represented is mean and s.e.m. (**c**) Real-time PCR was performed to validate the microarray with samples normalized to GAPDH as a loading control. *P<0.05, ***P<0.001; NS, not significant.

As PC is regularly diagnosed at advanced clinical stages, with over 50% of patients presenting with metastatic disease,^1^ we evaluated whether CaSm overexpression, which is believed to occur early in neoplastic progression, contributes to cellular migration and invasion. After 4 h, CaSm overexpression significantly increased transwell migration by >2-fold (Figure 4a). Similarly, invasion was analyzed using Matrigel-coated transwell chambers where, after 48 h, CaSm-overexpressing cells were nearly four times more invasive than driver controls (Figure 4b). Consistent with these observations, microarray results identified changes in genes associated with invasion and metastasis, notably increased mRNA expression of MMP1 and uPAR and decreased expression of SerpinB5 and NME1 (Figure 3a). Supporting the array conclusions, we confirmed that induced CaSm overexpression resulted in increased MMP1 expression (2.3-fold increase) and uPAR (8.2-fold increase) and decreased SerpinB5 (2.3-fold decrease); we did not, however, observe a statistical difference in NME1 expression between the two cell lines following real-time PCR validation (Figure 4c). This evidence suggests that CaSm expression may contribute to a metastatic phenotype and PC progression through altered genetic expression. To assess whether CaSm expression correlates with our genes of interest in human disease, we reviewed the Oncomine database to evaluate arrays from two pancreatic cancer investigations.^20, 21^ Each identified correlations between CaSm expression and several of the genes reported here (Supplementary Figure 5). Notably, both studies found that CaSm expression correlated with Slug, MMP1 and uPA/uPAR signaling in the patient pancreas samples. Although not conclusive, the data support that CaSm contributes to the expression of these critical genes.

### Increased CaSm promotes EMT

**Figure 5:**
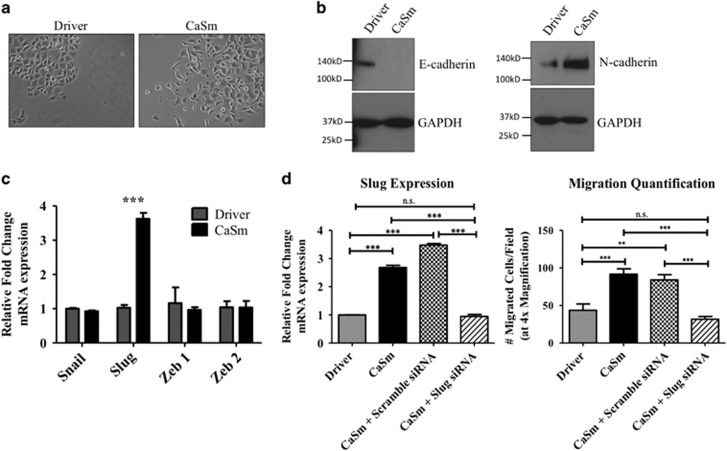
Overexpression of CaSm promotes EMT transition. (**a**) Tet-on driver and tet-on CaSm cells were grown in 1 μg/ml doxycycycline. Images were captured at × 100 magnification by Qcapture pro software (Surrey, BC, Canada). (**b**) E- and N-cadherin expression was assessed using whole-cell lysates from tet-on driver and tet-on CaSm Panc-1 cells separated on an 8% polyacrylamide gel with GAPDH used as loading control. (**c**) A panel of EMT transcription factors was evaluated by real-time PCR comparing expression between tet-on driver and tet-on CaSm Panc-1 cells grown under chronic doxycycline culture conditions (1 μg/ml). (**d**) Tet-on driver and tet-on CaSm cells were maintained with 1 μg/ml doxycycline before tet-on CaSm cells were either left untreated or transfected with 5 μg Slug or Scramble siRNA using Lipofectamine RNAi Max reagent. Cells were harvested 72 h after transfection for RNA isolation and real-time PCR (left) or plated for 4-h migration assay as previously described (right). Shown is a representative of three independent experiments with mean and s.e.m. depicted. **P<0.005, ***P<0.001, NS, not significant (*P*>0.05).

EMT is associated with metastasis in several cancers, as well as decreased chemosensitivity.^22, 23, 24^ Given that CaSm overexpression promoted resistance against gemcitabine therapy as well as enhanced cellular invasion and migration, we hypothesized that CaSm induction resulted in changes characteristic of EMT, such as cadherin switching and changes in transcription factor expression. When plated at low cellular densities, the tet-on CaSm Panc-1 cells obtained a more mesenchymal phenotype as compared with the driver controls (defined by increased spindle formation and decreased cellular adhesion; Figure 5a). CaSm induction also transformed the cadherin expression of the Panc-1 cells, resulting in a loss of epithelial E-cadherin protein and a gain of mesenchymal N-cadherin protein relative to the driver controls (Figure 5b). We performed real-time PCR to assess expression of EMT-associated transcription factors. Although expression of Snail, Zeb1 and Zeb2 was similar between tet-on driver cells and tet-on CaSm cells, Slug was significantly increased in the CaSm-overexpressing Panc-1 cells (Figure 5c), suggesting that the CaSm-mediated EMT phenotype may be due to Slug overexpression. To further evaluate this hypothesis, we used small interfering RNA (siRNA) to reduce Slug expression in the tet-on CaSm Panc-1 cells to levels equal to those observed in the driver control cells (Figure 5d). Seventy-two hours after siRNA treatment, cells were harvested for transwell migration assay as performed previously. Although untreated and scramble tet-on CaSm Panc-1 cells demonstrated high migratory potential after 4 h, the magnitude of migrated cells was reduced by twofold in the Slug knockdown cells (Figure 5d).

### CaSm induction promotes tumor formation and metastasis *in vivo*

**Figure 6:**
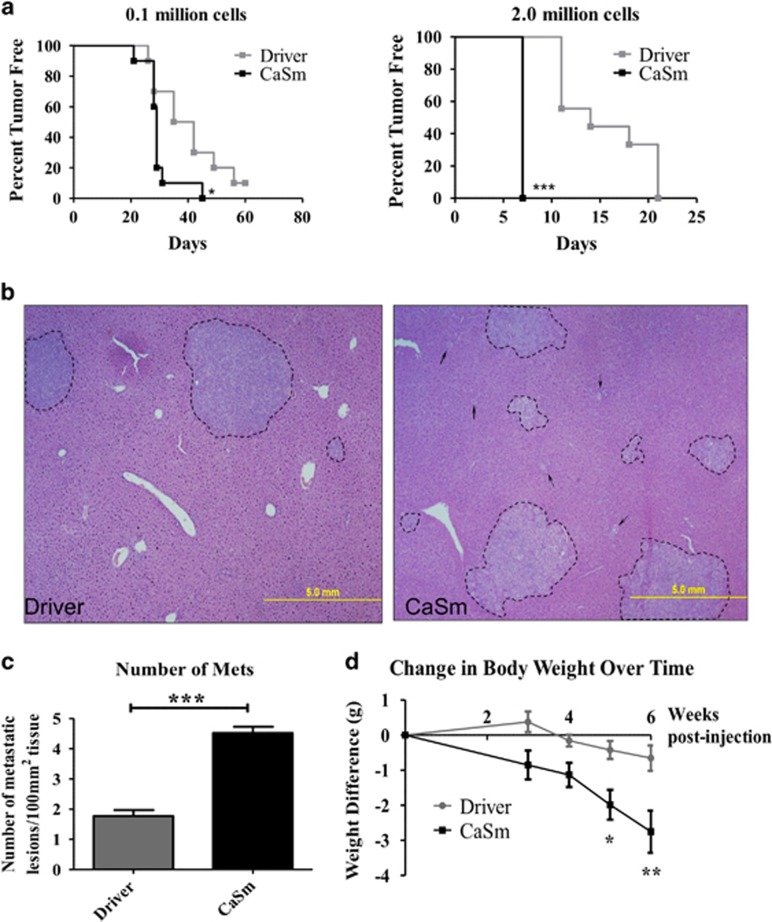
Induced CaSm overexpression enhances tumor formation and metastasis in NSG mice. (**a**) Tet-on driver and tet-on CaSm Panc-1 cells were maintained in chronic doxycycline (1 μg/ml) for 2–3 weeks before to subcutaneous injection of 0.1 × 10^6^ (*n*=10 for both tet-on driver and tet-on CaSm) or 2 × 10^6^ (*n*=9 tet-on driver and *n*=10 tet-on CaSm) cells on day 0. Expression was induced with 625 mg/kg doxycycline feed. Tumor presence was evaluated twice weekly by palpation beginning at day 7. Depicted is the accumulation of two independent experiments. (**b**–**d**) Tet-on driver and tet-on CaSm Panc-1 cells were maintained in chronic doxycycline (1 μg/ml) for 3 weeks before splenic injection on day 0 (*n*=6 driver, and *n*=6 CaSm). Expression was induced with 625 mg/kg doxycycline feed and animals were weighed weekly starting 3 weeks post-injection. After 6 weeks, animals were killed and livers and spleens were resected. Liver tissue was fixed in 10% formalin overnight and sectioned (one section cut every 500 μm, for five total sections per animal). Sections were stained for hematoxylin and eosin (H&E) (representative images, **b**) and assessed for the number of metastatic lesions/100 mm^2^ total tissue (**c**) by ImageJ, with at least five images per section assessed at × 40 magnification. Lesions with a diameter of at least 0.5 mm were quantified. (**d**) Animal weight was measured weekly beginning at week 3 post-injection. Shown here is the average weight loss compared with individual pre-injection weight. *P<0.05, **P<0.005, ***P<0.001.

Based on evidence suggesting that CaSm upregulation mediated EMT, a phenomenon also associated with increased tumorigenicity,^25, 26^ we next evaluated whether CaSm overexpression increased *in vivo* tumor-initiating capability. Immunocompromised NOD Scid Gamma (NSG) mice received subcutaneous dorsal injections of 1 × 10^5^ and 2 × 10^6^ tet-on driver or tet-on CaSm cells and tumor presence was examined biweekly for 8 weeks by palpation. Although nearly all of the mice established tumors during the course of the study, the tet-on CaSm cells formed tumors more readily at both cell concentrations (Figure 6a), demonstrating that induced CaSm expression was associated with enhanced tumor formation in these mice. By day 7, 100% of mice injected with 2 million tet-on CaSm Panc-1 cells formed palpable tumors, however, complete tumor uptake of the tet-on driver Panc-1 cells did not occur until day 21 (Figure 6a). Similarly, the median tumor-free survival for mice injected with 0.1 million tet-on driver cells was 38.5 days compared with just 29 days for the tet-on CaSm Panc-1 cells. These data support the premise that induced CaSm expression is associated with enhanced tumor formation *in vivo*.

Metastatic potential of the tet-on driver and tet-on CaSm cells was evaluated using our previously described murine model^27^ in NSG mice. Six weeks following splenic tumor cell injection, we evaluated the resulting hepatic metastases by using histological hematoxylin and eosin staining (8 μm sections every 500 μm of liver tissue for a total of five sections per animal) to characterize and quantify the metastatic hepatic lesions. Histological analysis showed differences in growth patterns of the liver lesions between the two conditions; whereas the driver controls resulted in tight and condensed lesions, the tet-on CaSm cells grew into more diffuse tumors with additional single-cell or small-cell clusters throughout the liver tissue (Figure 6b). Although there was no difference in primary tumor burden (Supplementary Figure 4), when normalized to total liver tissue area, tet-on CaSm Panc-1 cells produced significantly more metastatic lesions (4.523±0.21 tumors per 100 mm^2^ vs 1.775±0.20 tumors per 100 mm^2^ in the driver controls; Figure 6c), revealing that CaSm expression contributes to PC metastasis *in vivo*.

Interestingly, mice injected with tet-on CaSm Panc-1 cells demonstrated significantly greater weight loss over the course of the study compared with mice injected with the control tet-on driver Panc-1 cells; by the time of killing, mice injected with the tet-on driver Panc-1 cells had lost an average of 0.65 g in body weight, whereas the mice injected with tet-on CaSm Panc-1 cells lost an average of 2.1 g (Figure 6d), implying a previously unseen role for CaSm overexpression in cancer-related cachexia. Overall, we observed that induced CaSm overexpression enhanced the metastatic capability of the Panc-1 cells.

## Discussion

Our laboratory group previously identified CaSm as an oncogene whose overexpression is necessary for cellular transformation in pancreatic cancer and which has potential as a therapeutic target enhancing gemcitabine therapy.^14, 16, 17, 28^ A more specific understanding of CaSm's position in the development of PC remained largely unknown. Observations that induced CaSm overexpression resulted in increased cellular growth and clonogenic formation *in vitro*, along with previous reciprocal studies investigating the effects of CaSm knockdown, confirmed that upregulation of CaSm is an important contributor to cellular transformation in the development of PC.

Cell cycle analysis and SRB assays demonstrated that CaSm upregulation confers protection to Panc-1 cells against gemcitabine-induced cytotoxicity. Despite the observation that gemcitabine causes similar cell cycle arrest in the tet-on driver and tet-on CaSm cells, CaSm-induced Panc-1 cells are still differentially resistant to gemcitabine-induced cell death. Given that CaSm is essential in facilitating mRNA decapping and degradation,^10, 11, 12, 13^ we hypothesized that CaSm overexpression could alter the expression of key apoptotic regulators as a potential mechanism of gemcitabine resistance. The observed alterations in Bcl-X_L_, Bad and E2F1 expression following CaSm induction provide a possible mechanism behind CaSm-mediated chemoresistance, particularly as these genes have been previously implicated in gemcitabine resistance in PC and other cancers.^29, 30, 31^ Further work is needed to determine whether the addition of Bad and E2F1 or reduction of Bcl-X_L_ can rescue gemcitabine sensitivity in the CaSm-overexpressing Panc-1 cells.

In addition to building upon previous reports that CaSm is involved in proliferation,^14, 15, 17, 28^ transformation^14, 15, 17, 28^ and chemosensitivity,^18^ we also sought to investigate the novel hypothesis that CaSm promotes metastasis and invasion. Two studies have previously implicated contradicting roles for CaSm in metastatic regulation in other cancer pathologies *in vivo*. The first study, using human primary and metastatic prostate samples, concluded that Lsm1/CaSm serves as a metastasis suppressor in prostate cancer samples.^32^ The investigators also noted that stable Lsm1 transfection in PC3 cells did not increase proliferation or invasion *in vitro*.^32^ These results are directly at odds with our work and difficult to rationalize given not only the data presented here but also in the context of other investigations supporting that CaSm overexpression promotes a tumorigenic phenotype in many cancer pathologies, including prostate cancer.^14^ Investigation in human pancreatic endocrine tumors supported that CaSm may be involved in metastatic disease, with approximately half of primary tumors (7/15, 47%) and the majority of metastatic liver lesions (5/7, 71%) overexpressing CaSm mRNA compared with normal islet cells.^33^ Despite this, a direct association linking CaSm overexpression and metastatic pathways was previously unexplored. The data presented here support the possibility that CaSm mediates metastasis through Slug overexpression, which was demonstrated to have a direct role in the migratory capabilities of the CaSm-induced Panc-1 cells. Alterations in MMP1, uPAR and SerpinB5 expression, all of which are associated with invasion and metastasis in PC,^34, 35, 36, 37, 38, 39^ may also explain the observed CaSm-enhanced invasion and migration. Additional investigation is necessary to determine whether CaSm directly affects the RNA stability of MMP1, Slug, uPAR and SerpinB5 and to further assess the necessity of each of those genes in our observed phenotypes. Also, given that Slug knockdown reduced cellular migration to baseline control levels, it should be investigated whether MMP1, SerpinB5 and uPAR individually contribute to migration and invasion and whether these changes in expression are directly facilitated by CaSm or perhaps downstream of CaSm-mediated Slug overexpression. Provided that Slug expression correlated with CaSm in both PC patient arrays and given the efficacy of Slug siRNA to reduce CaSm-mediated migration, it would also be interesting to investigate using this gene as a therapeutic target to prevent metastatic disease.

Given the putative function of CaSm in RNA decapping and stability, it is not surprising that microarray results showed that CaSm induction affected expression within multiple cancer-associated pathways such as apoptosis and invasion. Additional cancer-related pathways that appear to be altered following CaSm induction include transforming growth factor-β, tumor necrosis factor and vascular endothelial growth factor, which are known to contribute to EMT/metastasis, cachexia and angiogenesis, respectively. Further work is needed to validate these genetic alterations and evaluate their relative importance in our functional observations. Previous studies of CaSm upregulation in a breast cell line demonstrated similar results with altered expression of genes associated with apoptosis, mitogen-activated protein kinase and transforming growth factor-β signaling pathways.^15^ Interestingly, there is no overlap comparing individual genes between the two studies,^15^ possibly due to the limited focus of our study or due to differences in expression between breast and pancreatic cancer cells.

CaSm was ubiquitously expressed in all of our human PC cell lines, attesting to its importance in PC development while limiting our experiments to the Panc-1 cells, which comparatively expressed the lowest amounts of CaSm protein. This work builds on previous studies investigating the effect of CaSm knockdown in human (AsPC-1 and Capan-1) and murine (Panc02) pancreatic cancer cell lines, in addition to studies in prostate and lung cancer cell lines.^14, 16, 17, 18, 28^ Studies in MCF10a and Sum44 human mammary cell lines further support that CaSm overexpression promotes proliferation and transformation, potentially through modification of cancer-related gene expression.^15^ In addition to this cell-based data, human pancreatic cancer microarray data indicate that CaSm expression correlates with several of the genes discussed here, notably Slug, MMP1 and uPAR signaling pathways. It is interesting to note that in both patient-based arrays, CaSm expression correlated with SerpinB5; whereas this contradicts the inverse relation identified through the microarray, SerpinB5 is commonly upregulated in pancreatic cancers,^40^ although known to be absent or downregulated in human breast, prostate and gastric cancers,^41^ indicating that, in addition to CaSm, other cancer-related pathways are likely involved in regulating expression of SerpinB5 in human pancreatic cancer.

Importantly, we were able to confirm that the molecular changes associated with upregulation of CaSm significantly contributed to tumor formation and dissemination of the Panc-1 cells *in vivo*, demonstrating that CaSm overexpression, believed to be an early event in PC progression, contributes to the highly metastatic and deadly nature of the disease. In addition, mice injected with tet-on CaSm cells presented with significant weight loss compared with mice injected with driver controls, further indicating that CaSm contributes to metastatic development, as cachexia indicates advanced metastatic disease and poorer prognosis in PC.^42, 43^

These studies provide important insight on the role of CaSm-mediated neoplastic progression and present potential pathways for the use of CaSm as a therapeutic agent. The pluripotent changes resulting from CaSm overexpression are consistent with current mechanistic evidence, which implies that CaSm is involved in facilitating mRNA decapping and degradation, with altered CaSm expression levels having the ability to lead to alterations in mRNA expression. Taken together, this information may be used to improve CaSm knockdown gene therapy or to find alternative measures to combat CaSm-mediated tumor progression in pancreatic cancer.

## Materials and methods

### Cells

Human PC cells were obtained from ATCC (Manassas, VA, USA) and maintained in RPMI (ASPC-1, BxPC3, Capan-2) or Dulbecco's modified Eagle's medium (DMEM) (HPAC, MiaPaCa-2, Panc-1) supplemented with 10% fetal bovine serum (FBS) (Hyclone, Logan, TX, USA) at 37 °C in 5% CO_2_. Cells were regularly screened for mycoplasma contamination using the MycoAlert Detection Kit (Lonza, Basel, Switzerland).

### CaSm inducible system

The full-length human CaSm DNA sequence was cloned into pRetro-X-Tight-Pur plasmid (Clontech, Mountain View, CA, USA). Panc-1 cells were transfected with the pRetro-X Tet-on-Advanced plasmid (Clontech) using Lipofectamine2000 (Invitrogen, Carlsbad, CA, USA) according to the manufacturer's protocol. Stable clones and pools were selected by G418 antibiotic resistance (1 mg/ml, Invitrogen) in complete media and then tested for doxycycline inducibility following a transient transfection with the pRetro-X-Luc control vector. The pool with the highest induction was used as the driver control cell line. A subset of this pool was co-transfected with the pRetro-X-Tight-Pur-CaSm plasmid and a stable pool obtained by G418 and puromycin (1 μg/ml, Invitrogen) antibiotic selection. CaSm expression was confirmed using real-time PCR and western blot, demonstrating low basal levels and efficient induction of the pool. Tet-on driver Panc-1 cells were maintained in DMEM media supplemented with 10% tetracycline-screened FBS (Hyclone), 1 mg/ml G418, and 1 μg/ml doxycycline (Sigma, St Louis, MO, USA). Tet-on CaSm Panc-1 cells were propagated in DMEM media supplemented with 10% tetracycline-screened FBS, 1 mg/ml G418, 1 μg/ml puromycin and 1 μg/ml doxycycline. Unless otherwise noted, experiments were initiated using cells grown in doxycycline-containing media for 4–9 weeks.

### Western blot analysis

Whole-cell lysates were obtained using RIPA buffer (ThermoScientific, Waltham, MA, USA) and protease inhibitors (Sigma). Protein (25–30 μg) was separated on a 12.5% polyacrylamide gel before being transferred to a Hybond ECL nitrocellulose membrane (Amersham, Pittsburgh, PA, USA). The following primary antibodies were used for overnight incubations, diluted in 5% non-fat milk: anti-CaSm IgY (2 μg/ml, as previously described),^28^ anti-E-cadherin (1 μg/5 ml, BD Biosciences 610181, San Jose, CA, USA) and anti-N-cadherin (10 μg/2 ml, Invitrogen 33–3900). Anti-GAPDH (1 μg/5 ml, Abcam 9485, Cambridge, MA, USA) was used as loading control. The membrane was incubated in horseradish peroxidase-conjugated secondary antibodies for 1 h. Goat-anti-IgY (1 μg/5 ml, Aves Labs, Portland, OR, USA) was used as secondary antibody against the CaSm antibody, horse-anti-mouse IgG (1 μg/5 ml, Cell Signaling Technology 7076, Boston, MA, USA) against the E- and N-cadherin antibodies, and goat-anti-rabbit-IgG (1 μg/5 ml, Abcam 6721) against the GAPDH antibody. West Pico Chemiluminescent reagent (Pierce, Rockford, IL, USA) was used to develop the membrane that was then was exposed to CL-Xposure film (ThermoScientific).

### Trypan blue cell counts

For cell growth assays, 100 000 tet-on driver and tet-on CaSm Panc-1 cells were plated in triplicate in six-well plates on day 0. On days 1, 3 and 4, cells were trypsinsized, neutralized with FBS-containing media, stained with 0.4% Trypan blue solution (Sigma) and quantified by hemocytometer (with at least 95% viability).

### Clonogenic assays

Fifty or 100 tet-on driver and CaSm Panc-1 cells were plated in single-cell suspension in triplicate in six-well plates. Media was refreshed twice weekly. After 14 days in culture, cells were fixed in 10% buffered formalin and stained with 2.5 mg/ml crystal violet in 2% ethanol. The number of colonies with >50 cells was recorded.

### Soft agar anchorage-independent growth assays

A 0.6% agar base was applied to six-well plates and allowed to solidify. Tet-on driver and tet-on CaSm Panc-1 cells were suspended in 0.4% agar in DMEM media supplemented with 1 μg/ml doxycycline and appropriate selection antibiotics and overlaid atop the base layer. Cells were plated at concentrations of either 500 or 1000 cells per well and fed weekly with 0.4% agar containing media and appropriate selection antibiotics. After 5 weeks, cells were stained with 2.5 mg/ml crystal violet in 2% ethanol for 1 h and destained with water. Colonies >0.1 mm were counted in triplicate wells.

### SRB assay

Tet-on driver or CaSm Panc-1 cells were seeded in each well of a 96-well plate (5000 cells per well) and incubated overnight. Cells were then treated with 0–100 μM gemcitabine (Gemzar, Eli Lilly, Indianapolis, IN, USA) with replicates six wells per condition. Ninety-six hours after treatment, cells were fixed with 5% trichloroacetic acid (Sigma) for an hour, stained with 0.4% SRB (Sigma) for 30 min and dissolved in 10 mM Tris Base before absorbance was read at 560–564 nm. Percent survival was determined by calculating the absorbance of treated cells to respective untreated controls.

### Cell cycle

Tet-on driver and tet-on CaSm Panc-1 cells were seeded overnight in duplicate in a six-well plate (100 000 cells per well) before being treated with 200 nM gemcitabine. Cells were harvested on days 0, 1, 3 and 5 and fixed in ice-cold 70% ethanol overnight before resuspension in 0.1 μg/μl propidium iodide (Sigma) and 0.2 μg/μl RNase A (Sigma) followed by flow cytometry cell cycle analysis with the LSRFortessa (BD, Franklin Lakes, NJ, USA). Apoptosis was determined as the percentage of cells with sub-G1 content.

### Microarray analysis

Tet-on driver and tet-on CaSm Panc-1 cells were grown with and without doxycycline (1 μg/ml) for 4 weeks. Levels of CaSm expression were confirmed using reverse transcriptase–PCR (Supplementary Figure 2a). RNA isolation was performed using RNeasy Plus kit (Qiagen, Valencia, CA, USA) following the manufacturer's protocols. The Hollings Cancer Center Tissue Biorepository core facility confirmed RNA quality using the 18S/28S Ratio Integrity Number using Agilent 2100 Bioanalyzer (Agilent Technologies, Santa Clara, CA, USA); RNA with a RIN of 10 was used for further analysis (Supplementary Figure 2b). Complement DNA synthesis was performed using RT^2^ First Strand kit and RT^2^ SYBR Green qPCR master mix was used for the microarray master mix reagent according to the manufacturer's manual (SABiosciences, Valencia, CA, USA). The Cancer PathwayFinder PCR array (SABiosciences) was run on the Roche 480 Lightcycler (Roche, Indianapolis, IN, USA) with two plates run for each tet-on driver+doxycycline, tet-on CaSm and tet-on CaSm+doxycycline. The plates included controls for genomic DNA contamination, reverse transcription and positive controls. Analysis was performed using SABioscience's Free PCR Array Data Analysis Software (ΔΔCt method) with B2M, HPRT1 and GAPDH as pooled housekeeping genes. The raw and normalized data are available on the GEO Omnibus repository (accession code GSE74319).

### Real-time PCR

RNA was isolated with the RNeasy Plus kit (Qiagen) and complementary DNA synthesis performed using iScript cDNA synthesis kit (Bio-Rad, Berkeley, CA, USA) with 1 μg of RNA template. Real-time PCR was performed with 2.5 μl of a 1:10 dilution of complementary DNA using 5 μl Lightcycler 480 Probes Master mix (Roche), 0.1 μl fluorescent probe (Roche) and 0.5 μM forward and reverse primers (Sigma). The following cycling conditions were used: pre-incubation at 95 °C for 10 min, 40 cycles of denaturing at 95 °C for 10 s, annealing at 55° for 10 s and extension at 72 °C for 30 s. A single data acquisition occurred at the end of each extension period. Relative expression was calculated using ΔΔCt, with values normalized to GAPDH.[Table tbl1]

### Migration assay

Twenty-four-well cell culture inserts with 8.0 um pores (BD Biosciences) were coated with 15 μg/ml fibronectin (ThermoScientific) overnight at 4 °C. Tet-on driver and tet-on CaSm Panc-1 cells were plated in serum-free DMEM onto the top chamber of the fibronectin-coated chambers (20 000 cells per well); DMEM supplemented with 10% FBS was used as the chemoattractant in the bottom wells. The migration chambers were incubated at 37 °C for 4 h. At this point, non-migrated cells were removed by gently wiping the top chamber three times with a clean cotton swab. Diff-Quick kit (Siemens, Malvern, PA, USA) was used to fix and stain the migrated cells. Cells were plated in triplicate and counted at × 40 magnification with at least five fields counted per chamber.

### Invasion assay

Invasion assays were performed using 24-well BioCoat Matrigel Invasion Chambers (BD) according to the manufacturer's instructions. Matrigel chambers were rehydrated as directed before plating 15 000 tet-on driver or tet-on CaSm Panc-1 cells in serum-free DMEM onto the top chamber the inserts; DMEM supplemented with 10% FBS was used as the chemoattractant in the bottom of the wells. After 48-h incubation at 37 °C, non-invading cells on the top of the insert membrane were gently scrubbed away before invasive cells were stained with Diff-Quick and counted at × 40 magnification. Cells were plated in triplicate with three fields counted per chamber.

### Slug siRNA studies

Tet-on driver and CaSm cells were plated in six-well plates and allowed to reach 40% confluence. Tet-on CaSm cells were either left untreated or transiently transfected with 5 μg scramble (Stealth RNAi Negative Control, Invitrogen) or Slug siRNA (Invitrogen HSS185949) using Lipofectamine RNAiMAX (Invitrogen). Seventy-two hours after transfection, cells were trypsinized for real-time PCR and migration assays as described above.

### Mice

Six-to-eight-week old NOD.Cg-*Prkdcscid Il2rgtm1Wjl*/SzJ (NSG) mice were purchased from Jackson Labs (Bar Harbor, ME, USA) and maintained in DLAR facilities with food and water provided *ad libitum*. All murine experiments were performed according to protocols approved by the Institutional Animal Care and Use Committee at the Medical University of South Carolina.

### Subcutaneous murine injections

NSG mice received dorsal subcutaneous injections of 1 × 10^5^ (*n*=10 for both tet-on driver and tet-on CaSm) or 2 × 10^6^ (*n*=9 tet-on driver and *n*=10 tet-on CaSm) cells in 200 μl phosphate-buffered saline on day 0. Animals were randomly assigned to groups during the time of cell injections. Doxycycline feed (625 mg/kg, Harlan Laboratories, Indianapolis, IN, USA) was provided to all animals *ad libitum* beginning on day 0. Tumor presence was evaluated by palpation twice a week, beginning on day 7. All cells were expanded in the presence of 1 μg/ml doxycycline for 2–3 weeks before injection. No animals were excluded over the course of the study.

### Splenic murine injections

NSG mice were anesthetized using 2% isofluorane (Baxter, Deerfield, IL, USA) in 1.5 l/min oxygen and received a single, preemptive analgesic of 3 mg/kg carprofen (Sigma Aldrich, St Louis, MO, USA). The left flank was shaved and sterilized with Betadine (Purdue Products, Stamford, CT, USA). Once appropriate anesthesia was achieved, 1 cm left subcostal incision was made through the abdominal skin and peritoneum. Tet-on driver or tet-on CaSm Panc-1 cells in (1 × 10^6^ cells in 0.05 ml phosphate-buffered saline (Hyclone)) were injected into the exteriorized spleen using a sterile 28-gauge needle and hypodermic syringe. Animals were randomized into groups at the time of injection (*n*=6 for each tet-on driver and tet-on CaSm Panc-1 cells). Cells had previously been maintained in 1 μg/ml doxycycline for 3 weeks before injection. Doxycycline feed (625 mg/kg, Harlan Laboratories) was provided *ad libitum* to all animals beginning on day 0. Animal weights were taken at the time of tumor injection and weekly beginning at week 3; no animals were excluded over the course of the study. Six weeks after injection, mice were killed and livers and spleens resected. Liver tissue was fixed overnight in 10% buffered formalin, set in paraffin blocks and cut into 8 μm thick sections (Tissue Biorepository Core at the Hollings Cancer Center, Medical University of South Carolina), with one section cut every 500 μm for five total sections per sample. Sections were stained with standard hematoxylin and eosin staining by the Tissue Biorepository Core facility. Hematoxylin and eosin images were analyzed at × 40 magnification with at least five fields per section. Tumor area was quantified on the images using ImageJ software (National Institutes of Health, Bethesda, MD, USA). The number of hepatic lesions/image was evaluated and normalized to the total tissue area of the image by a blinded investigator.

### Statistical analysis

Statistical analysis was performed using GraphPad Prism 5 software (La Jolla, CA, USA). Proliferation and SRB assays were analyzed using two-way analysis of variance, and tumor-free survival was analyzed using the log-rank Mantel Cox test. Two-tailed unpaired Student's *t*-tests were used for statistical analysis in all other experiments. For the subcutaneous cell injections, a sample size of at least nine animals per group was needed to achieve 80% power, assuming that the tet-on CaSm cells would form tumors twice as readily as the tet-on driver cells (day 7 vs day 14, with a 5-day s.d.). For the metastatic model, at least four animals per group were needed to achieve 80% power, assuming that the tet-on CaSm cells would form twice the number of liver metastases as the tet-on driver controls (4.0±1.0 mets/100 mm^2^ compared with 2.0±1.0 mets/100 mm^2^). In all studies, *P*-values <0.05 were considered significant.

## Figures and Tables

**Table tbl1:** Primer sequences are as follows:

*Gene*	*UPL probe*	*Forward primer*	*Reverse primer*
Bad	# 45	5′-accagcagcagccatcat-3′	5′-ggtaggagctgtggcgact-3′
Bcl-XL	# 66	5′-agccttggatccaggagaa-3′	5′-agcggttgaagcgttcct-3′
CaSm	# 82	5′-ttcctcgagggatttttgtg-3′	5′-tacttgctggaggggtgtg-3′
E2F1	# 5	5′-tccaagaaccacatccagtg-3′	5′-ctgggtcaacccctcaag-3′
GAPDH	# 60	5′-gctctctgctcctcctgttc-3′	5′-acgaccaaatccgttgactc-3′
FAS	# 65	5′-atggccaatctgccataag-3′	5′-tgactgtgcagtccctagctt-3′
MMP1	# 7	5′-tgagggtcaagcagacatca-3′	5′-ctggttgaaaagcatgagca-3′
NME1	# 11	5′-cagccggagttcaaacctaa-3′	5′-gcaatgaaggtacgctcaca-3′
SerpinB5	# 88	5′-catgttcatcctactacccaagg-3′	5′-tctgagttgagttgtttttcaatctt-3′
Slug	# 7	5′-tggttgcttcaaggacacat-3′	5′-gttgcagtgagggcaagaa-3′
Snail	# 11	5′-gctgcaggactctaatccaga-3′	5′-atctccggaggtgggatg-3′
uPA	# 46	5′-ttgctcaccacaacgacatt3′	5′-ggcaggcagatggtct-3′
uPAR	# 1	5′-acaccaccaaatgcaacga-3′	5′-ccccttgcagctgtaacac-3′
Zeb1	# 57	5′-aactgctgggaggatgacac-3′	5′-tcctgcttcatctgcctga-3′
Zeb2	# 16	5′-caaaaacctcgccaagagtg-3′	5′-ttcagggtctctcgtctctctc-3′
