# Supplementary figures and images for: The CaSm (LSm1) oncogene promotes transformation, chemoresistance and metastasis of pancreatic cancer cells
Source: Oncogenesis. 2016 Jan 11;5(1):e182–. doi: 10.1038/oncsis.2015.45 (PMC4728675; doi:10.1038/oncsis.2015.45)

***ng/mL Dox***

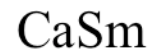

GAPDH

### *Hours Post-Dox*

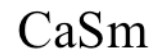

GAPDH

Supplement: Supplementary Figure 1 [file oncsis201545x1.pdf]

## A. Real-Time PCR Prior to Array

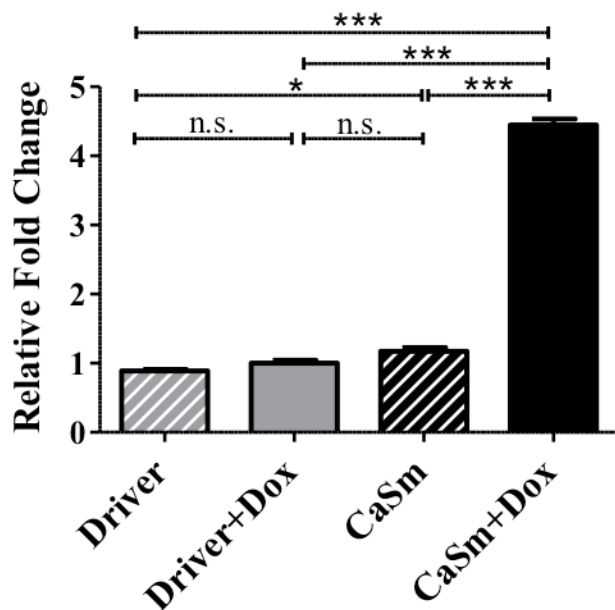

## B.

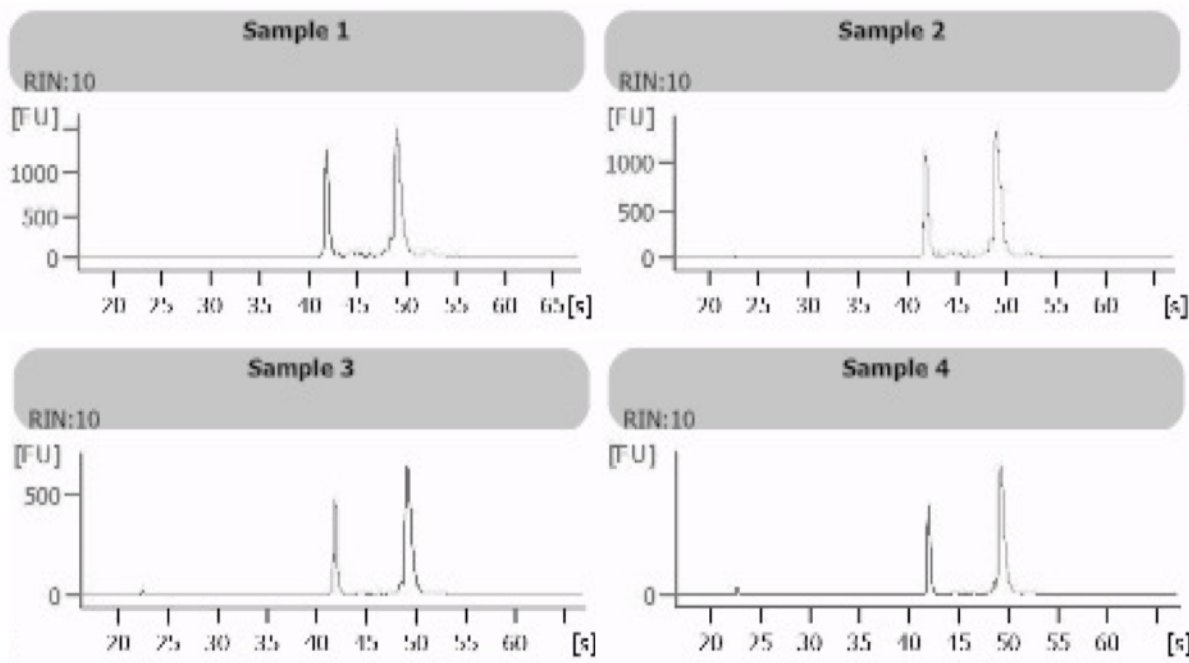

Supplement: Supplementary Figure 2 [file oncsis201545x2.pdf]

# Spleen Weight

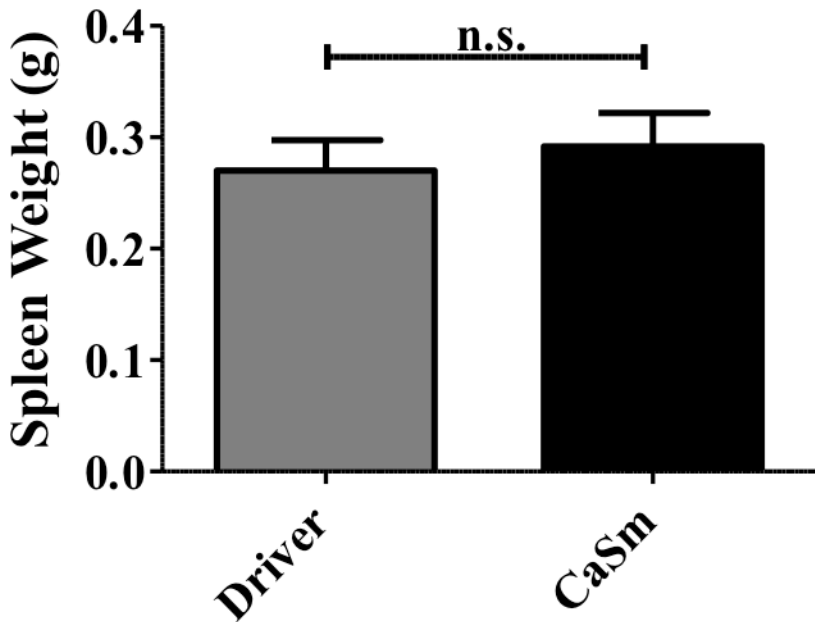

Supplement: Supplementary Figure 4 [file oncsis201545x4.pdf]

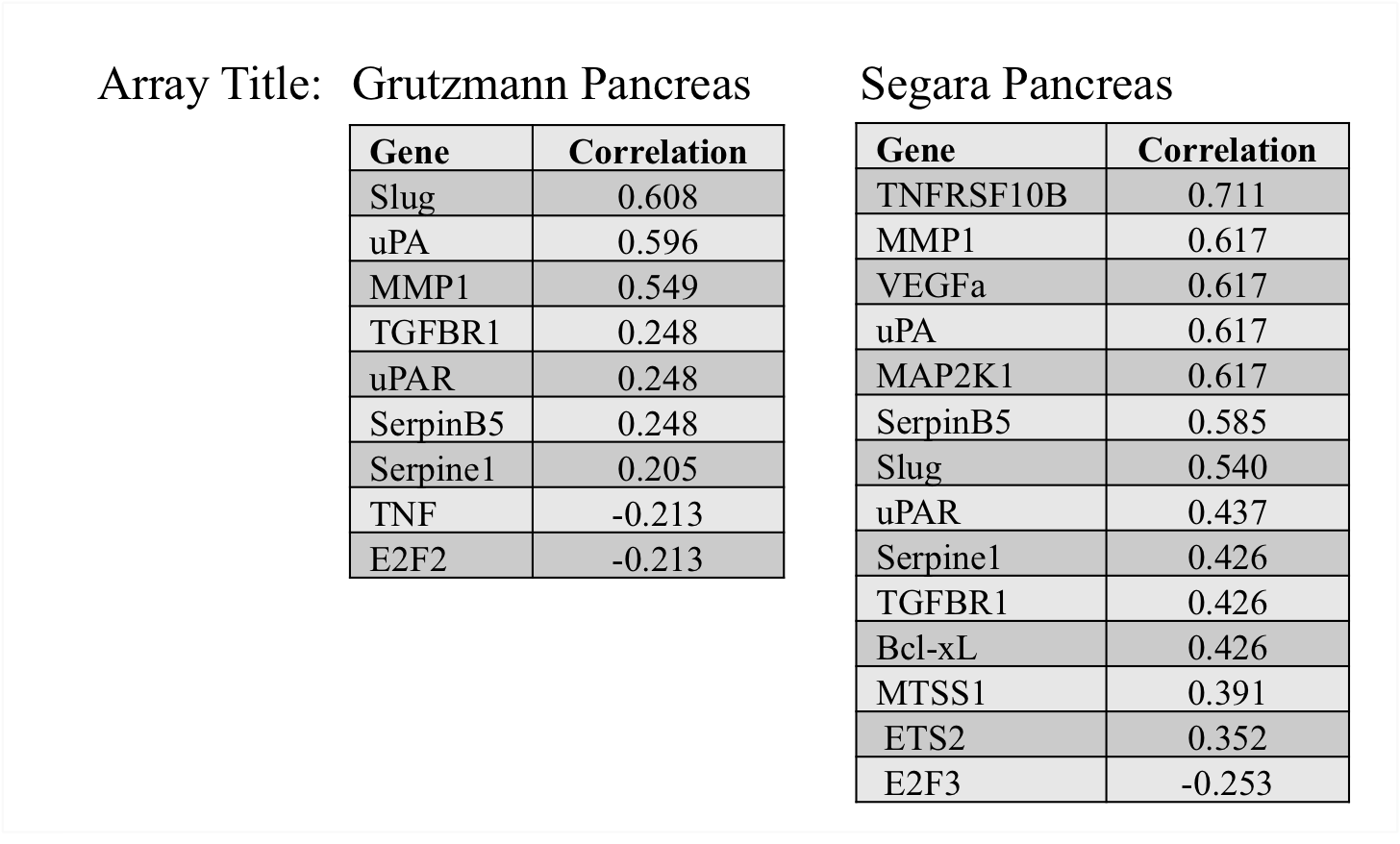

Supplement: Supplementary Figure 5 [file oncsis201545x5.png]
