# Supplementary material for: The CaSm (LSm1) oncogene promotes transformation, chemoresistance and metastasis of pancreatic cancer cells
Source: Oncogenesis. 2016 Jan 11;5(1):e182–. doi: 10.1038/oncsis.2015.45 (PMC4728675; doi:10.1038/oncsis.2015.45)
Supplement: Supplementary Figure 3 [file oncsis201545x3.pdf]

Gene	Fold-Change	P-value
Bad	-2.19	0.0062
Bcl-xL	1.35	0.3393
E2F1	-2.36	0.0278
ETS2	-2.78	0.0028
Fas	-1.74	0.1847
ITGA2	-1.86	0.0021
ITGB3	2.01	0.0247
MAP2K1	1.4	0.0049
MMP1	1.77	0.0090
MTSS1	2.46	0.0287
MYC	2.14	0.0730
NME1	-1.87	0.0018
uPAR	1.94	0.1263
SerpinB5	-1.28	0.1411
Serpine1	-1.96	0.0073
TGFBR1	3.76	0.1018
TNF	-1.01	0.9258
TNFRSF10B	2.15	0.0460
VEGFa	2.56	0.0453
B-Actin	-3.17	0.0001
