# Supplementary material for: The CaSm (LSm1) oncogene promotes transformation, chemoresistance and metastasis of pancreatic cancer cells
Source: Oncogenesis. 2016 Jan 11;5(1):e182–. doi: 10.1038/oncsis.2015.45 (PMC4728675; doi:10.1038/oncsis.2015.45)
Supplement: Supplementary Figure Legends [file oncsis201545x6.doc]

**Supplementary Figure 1. CaSm induction is doxycycline dose- and time- dependent.** Tet-on CaSm Panc-1 cells were treated with doxycycline (0-1 µg/mL for 24 hours panel A, 1 µg/mL for 0-24 hours panel B) before harvesting for whole cell lysate protein isolation and Western blot analysis to evaluate CaSm induction with GAPDH used a loading control.

**Supplementary Figure 2.** **Samples were analyzed for CaSm expression and RNA quality prior to PCR array.** A) Tet-on driver and CaSm cells were grown with or without 1 µg/mL doxycycline for 4 weeks prior to analysis of CaSm expression by real-time PCR, with all samples normalized to GAPDH and the tet-on driver + doxycycline control. B) RNA quality was determined by Ratio Integrity Number (RIN) using the Agilent 2100 bioanalyzer at the MUSC Hollings Cancer Center tissue biorepository, where sample 1 is tet-on CaSm; sample 2 tet-on CaSm + doxycycline; sample 3, tet-on driver; and sample 4, tet-on driver + doxycycline. ****P*<0.001, n.s. = not significant (*P*>0.05).

**Supplementary Figure 3. Microarray results comparing CaSm overexpression to all non-induced controls.** Tet-on driver and tet-on CaSm Panc-1 cells were maintained with or without 1 µg/mL doxycycline for 4 weeks before RNA expression was analyzed by the SABioscience Cancer PathwayFinder PCR-based array. Genes with a 1.5 fold or greater alteration are comparing means of two experimental (tet-on CaSm + doxycycline) arrays to four controls (2 tet-on CaSm – doxycycline, 2 tet-on driver + doxycycline) are depicted.

**Supplementary Figure 4. Spleen primary tumor burden.** Tet-on driver and tet-on CaSm Panc-1 cells were maintained in chronic doxycycline (1μg/mL) for 3 weeks prior to splenic injection on day 0 (n = 6 driver, and n = 6 CaSm). Expression was induced with 625 mg/kg doxycycline feed. After 6 weeks, animals were sacrificed and spleens were resected and weighed as a measurement of primary tumor burden. n.s. = not significant (*P*>0.05)

***Supplementary Figure 5. CaSm expression correlates with our genes-of-interest in patient pancreas arrays.***  The Oncomine database (Compendia Bioscience, Ann Arbor, MI) was reviewed to evaluate the coexpression of CaSm (LSm1) and our genes of interest in two patient microarrays (Human Genome U133A Array). The Grutzmann array evaluated 17,779 genes among normal pancreas (n=11) and pancreatic adenocarcinoma (n=14) samples . The Segara array evaluated 12, 624 genes among normal pancreas (n=6) and pancreatic carcinoma (n=11) samples . Data mining correlation was calculated by Oncomine using hierarchical clustering as further described on the Oncomine webpage.
